# High oil accumulation in tuber of yellow nutsedge compared to purple nutsedge is associated with more abundant expression of genes involved in fatty acid synthesis and triacylglycerol storage

**DOI:** 10.1186/s13068-021-01909-x

**Published:** 2021-03-02

**Authors:** Hongying Ji, Dantong Liu, Zhenle Yang

**Affiliations:** 1 Key Lab of Plant Resources, Institute of Botany, Chinese Academy of Sciences, Beijing, 100093 China; 2 University of Chinese Academy of Sciences, Beijing, 100049 China

**Keywords:** *Cyperus esculentus*, *Cyperus rotundus*, Tuber, RubisCO bypass, Fatty acid synthesis, Triacylglycerol storage, Oil accumulation, Transcriptional control

## Abstract

**Background:**

Yellow nutsedge is a unique plant species that can accumulate up to 35% oil of tuber dry weight, perhaps the highest level observed in the tuber tissues of plant kingdom. To gain insight into the molecular mechanism that leads to high oil accumulation in yellow nutsedge, gene expression profiles of oil production pathways involved carbon metabolism, fatty acid synthesis, triacylglycerol synthesis, and triacylglycerol storage during tuber development were compared with purple nutsedge, the closest relative of yellow nutsedge that is poor in oil accumulation.

**Results:**

Compared with purple nutsedge, high oil accumulation in yellow nutsedge was associated with significant up-regulation of specific key enzymes of plastidial RubisCO bypass as well as malate and pyruvate metabolism, almost all fatty acid synthesis enzymes, and seed-like oil-body proteins. However, overall transcripts for carbon metabolism toward carbon precursor for fatty acid synthesis were comparable and for triacylglycerol synthesis were similar in both species. Two seed-like master transcription factors ABI3 and WRI1 were found to display similar transcript patterns but were expressed at 6.5- and 14.3-fold higher levels in yellow nutsedge than in purple nutsedge, respectively. A weighted gene co-expression network analysis revealed that *ABI3* was in strong transcriptional coordination with *WRI1* and other key oil-related genes.

**Conclusions:**

These results implied that pyruvate availability and fatty acid synthesis in plastid, along with triacylglycerol storage in oil bodies, rather than triacylglycerol synthesis in endoplasmic reticulum, are the major factors responsible for high oil production in tuber of yellow nutsedge, and ABI3 most likely plays a critical role in regulating oil accumulation. This study is of significance with regard to understanding the molecular mechanism controlling carbon partitioning toward oil production in oil-rich tuber and provides a valuable reference for enhancing oil accumulation in non-seed tissues of crops through genetic breeding or metabolic engineering.

**Supplementary Information:**

The online version contains supplementary material available at 10.1186/s13068-021-01909-x.

## Background

Yellow nutsedge (*Cyperus esculentus* L.) is a grass-like C_4_ plant species of the sedge family (Cyperaceae). Its tuber can be eaten raw, dried, cooked, and roasted, or consumed in wheat-like flour and milky beverage forms [1, 2]. As an unconventional underground tuber crop, yellow nutsedge is quite unique, since it is the only one plant species so far that can accumulate oil at high levels in its tuber tissues (up to 35% of dry weight) [3], in striking contrast to common root/tuber crops such as potato, sweet potato, and sugar beet that have very low levels of oil, while store exclusively starch or sugars as the major reserves in their storage organs. Compared to conventional oilseed crops including soybean, rapeseed, and peanut, yellow nutsedge is endowed with many advantageous characteristics such as wide soil adaptability, strong fertility, short growth period, little maintenance during growth, unlikely to be susceptible towards disease and pest harm, and high tuber yield (up to 12 t ha^−1^) [4–6]. Even so, yellow nutsedge is still an underutilized and nonpopular oil crop around the world, probably because it is a non-traditional crop and harvesting such a crop is laborious. Moreover, the mechanism of oil accumulation in yellow nutsedge is largely unknown at the molecular level, which hinders its potential application and development in oil production.

Given significant amounts of all the major storage components, i.e., starch, oil, sugar, and protein, are produced in its tubers [2, 7, 8], yellow nutsedge is regarded as a novel model system to study carbon flux toward oil synthesis in underground storage tissues. Biochemical analysis revealed that the accumulation patterns of oil, starch and soluble sugars appeared in a sequential way in developing tuber [8], i.e., sugar loading was paralleled with the beginning of starch accumulation upon the initialization of tuber development, and followed by later occurrence of oil accumulation, which was accompanied by the significant decrease in sugar levels. However, it is still unclear how carbon is partitioned and how oil synthesis is directed and regulated in tuber. Currently, the scarcity of molecular resources and genomic information available for this crop also hinders our understanding of the underlying molecular mechanism governing oil production.

The synthesis of plant oil in the form of triacylglycerol (TAG) is well understood in oil-rich seed plants particularly in model plant Arabidopsis [9]. In general, oil synthesis mainly involves de novo synthesis of fatty acids in plastid and their sequential acylation of the glycerol backbone via acyl-CoA-dependent and -independent reactions in endoplasmic reticulum (ER). Although oil synthesis in oil-seed plants is well characterized and the core metabolic processes or pathways are considered to be conserved among diverse plants, the biochemical and molecular details occurring in underground oil-rich sink organs such as yellow nutsedge tuber remain largely unknown.

It is noted that a closely related species of yellow nutsedge, purple nutsedge (*Cyperus rotundus*), a traditional medicinal plant long used in several countries including China, India, and Japan to develop health and pharmacological products [10], contains less than 5% oil of dry weight in its tuber, while stores high amounts of carbohydrates (starch and sugar) [7, 11]. Moreover, the habit and habitat as well the growth and development of the two species are quite similar [12]. Thus, purple nutsedge provides a good reference for exploring the biological mechanism of why and how yellow nutsedge accumulates high amount of oil in tubers. To decipher the molecular basis controlling such considerable differences in oil content, and better understand the regulation mechanisms of oil accumulation in oil-rich tuber of yellow nutsedge, in this study we made a comparative analysis of the global gene expression related to oil accumulation in developing tubers of yellow nutsedge with purple nutsedge. Our results revealed that high oil accumulation in tuber of yellow nutsedge is tightly associated with more abundant transcripts for fatty acid synthesis and triacylglycerol storage as compared to purple nutsedge.

## Results

### Tuber oil contents differed markedly between yellow and purple nutsedge

Under our experimental conditions, new shoots from seed tubers of the two nutsedges appeared above soil at 5–9 days after sowing on April 16, 2016. New tubers began to appear from 6 to 8 weeks after shoot emergence. Both species lasted around 5 months for their growth and development.

**Fig. 1:**
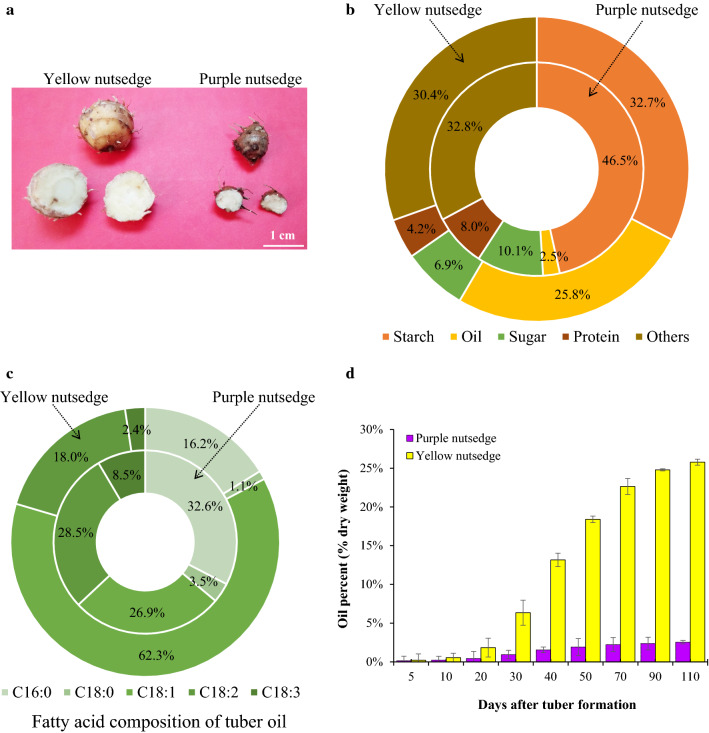
Proximate components of tubers from yellow and purple nutsedge. **a** Open tubers. **b** Proximate composition (as % dry weight) of mature tubers. **c** Fatty acid composition of oil in mature tubers. **d** Percentage of oil content on basis of tuber dry weight during tuber development. Values represent means ± SD (*N* = 3)

Analyses of the proximate constituents of mature tubers (Fig. 1a) indicated that there were marked differences between two species in the contents of major storage reserves including starch, oil, sugar, and protein on a tuber dry weight basis (Fig. 1b), where starch was the component at highest level in tubers of two species. The most notable difference was that yellow nutsedge stored more than 25% oil of dry weight in mature tubers, whereas purple nutsedge contained less than 3% oil, indicating that there is around tenfold difference in oil content.

Analysis of fatty acid composition of oil from mature tubers showed that yellow nutsedge predominated with oleic acid (C18:1) that accounted for more than 60% of total fatty acids, while purple nutsedge was represented with palmitic acid (16:0), C18:1, and linoleic acid (18:2) as major fatty acids, with concentrations ranging from 25 to 35% (Fig. 1c). These results indicated that significant difference also occurred in fatty acid composition of tuber oil between these two species, where purple nutsedge contained less oleic acid and more saturated fatty acids than yellow nutsedge.

To check whether there was also a difference in oil accumulation occurred in developing tubers, the changes in oil contents during tuber development were determined for two species. The oil accumulation patterns in the development period spanning around 110 days after tuber formation (DAF) are shown in Fig. 1d. The results indicated that the oil accumulation in the two types of tubers continued to increase throughout the tuber development. In all developmental stages, however, yellow nutsedge contained a significantly higher percentage of oil in tubers than purple nutsedge.

Overall, the striking differences in oil content and the fatty acid composition were present between these two types of tubers, suggesting that there existed distinct transcriptional control of oil production between the two species.

### Overall level of transcripts for oil production was higher in yellow nutsedge than in purple nutsedge

To uncover the difference in the transcriptional control of oil production between the two species, we systematically conducted comparative transcriptome analyses of oil-related genes in developing tubers. Tuber samples at three different developmental stages (i.e., the early stage 20 DAF, the middle stage 50 DAF and the late stage 90 DAF) were used for transcript analysis.

**Fig. 2:**
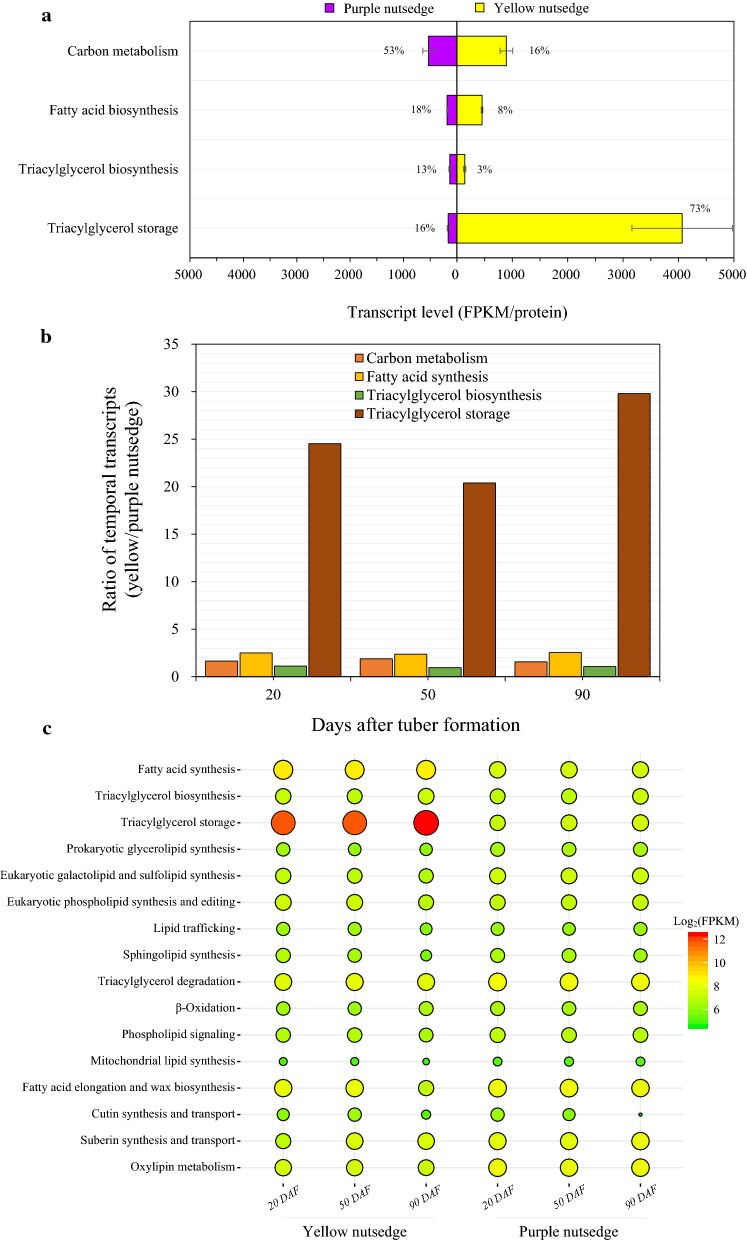
Expression pattern for the selected four pathways related to oil production in tubers. **a** The relative distribution (%) of transcripts among the four metabolic pathways from sucrose to TAG storage. Transcript level (FPKM/protein) of each pathway in two species was also displayed. The FPKM values for subunits of a protein or for multiple isoforms were summed. The data are averaged on all developing stages of tubers with error bars indicating their standard deviation. **b** Ratio of transcriptional levels between two species at three development stages*.* **c** Temporal transcripts for lipid-related metabolic pathways during tuber development. DAF, days after tuber formation

Oil production involves the conversion of sucrose up to TAG assembly or storage, primarily including cytosolic and plastidial carbon metabolism toward pyruvate generation in plastid, fatty acid (FA) synthesis in plastid, TAG synthesis in ER and TAG storage in oil body or lipid droplet. These four major metabolic processes were related to expression of more than 400 genes (Additional file 1: Tables S2 and S3). In this study, transcript levels were represented by FPKM (fragments per kilobase of exon model per million mapped reads) per protein, where multiple transcripts for genes encoding for isoforms or subunits of the same protein family were summed. Among these metabolic pathways, more than 70% of the transcripts in yellow nutsedge were associated with genes involved in TAG storage and only 8% and 3% were related to FA synthesis and TAG synthesis, respectively (Fig. 2a). In contrast, in purple nutsedge the transcripts related to carbon metabolism were most abundant, while those of TAG storage were the second least.

For every metabolic pathway, transcript levels were on average higher in yellow nutsedge than in purple nutsedge (Fig. 2a). The largest difference was noted for TAG storage, for which the transcript level was more than 20-fold higher in yellow nutsedge compared to purple nutsedge. Clear difference was also present for FA synthesis, where there was over two times higher in yellow nutsedge than in purple nutsedge. Unexpectedly, there was no substantial differences in carbon metabolism or TAG synthesis between two species. A similar contrast between the two plants also occurred across the three developmental stages of tubers (Fig. 2b). Notably, transcript patterns for other lipid-related metabolic pathways were comparable in two species (Fig. 2c).

Overall, the results mentioned above suggested that transcriptional control of genes involved in TAG storage along with FA synthesis rather than TAG synthesis might be the major factors required for high oil accumulation of yellow nutsedge in relative to purple nutsedge.

### Transcripts for carbon metabolism toward fatty acid synthesis were slightly higher in yellow nutsedge than purple nutsedge

**Fig. 3:**
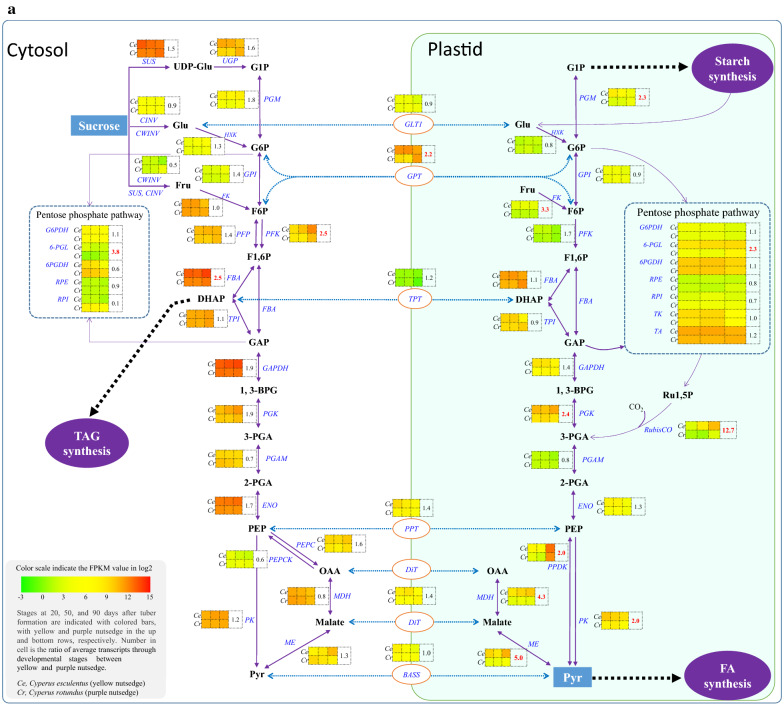

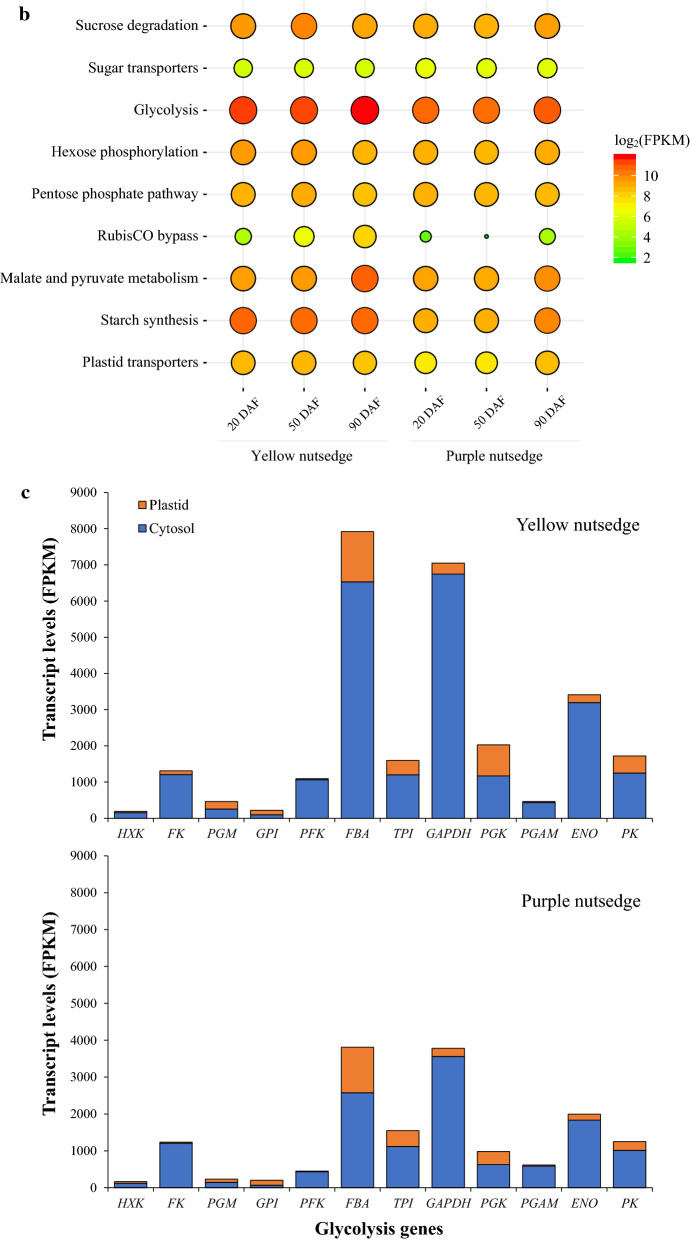
Transcript levels for carbon metabolism enzymes. **b** Temporal transcript levels for various carbon metabolic pathways or processes. Transcripts for proteins with isoforms or multiple subunits were summed. **a** Transcript patterns for genes involved in carbon metabolism. Gene names are indicated in blue. Value in table cell indicates the transcript ratio of yellow to purple nutsedge. Ratios without less than twofold are represented in red boldface. **c** Transcript levels for glycolysis enzymes in the cytosol and the plastid. 1,3-BPG, 1,3-Bisphosphoglycerate; 2-PGA, 2-phosphoglycerate; 3-PGA, 3-phosphoglycerate; 6PGDH, 6-phosphogluconate dehydrogenase; 6-PGL, 6-phosphogluconolactonase; BASS, sodium bile acid symporter family protein; CINV, cytosolic invertase; CWINV, cell wall invertase; DHAP, dihydroxyacetone phosphate; ENO, enolase; F1,6P, fructose 1,6 bis-phosphate; F6P, fructose 6-phosphate; FBA, fructose bisphosphate aldolase; FK, fructokinase; Fru, fructose; G1P, glucose 1-phosphate; G6P, glucose 6-phosphate; G6PDH, glucose 6-phosphate dehydrogenase; GAP, glyceraldehyde 3-phosphate; GAPDH, glyceraldehyde 3-phosphate dehydrogenase; GLT1, plastidic glucose translocator 1; Glu, glucose; HXK, hexokinase; MDH, malate dehydrogenase; ME, malic enzyme; NTT, nucleoside triphosphate transporter; OAA, oxaloacetate; PEP, phosphoenol pyruvate.; PEPC, phosphoenolpyruvate carboxylase; PEPCK, phosphoenolpyruvate carboxykinase; PFK, phosphofructokinase; PFP, pyrophosphate dependent phosphofructokinase; PGAM, 2,3-bisphosphoglycerate-dependent phosphoglycerate mutase; PGI, phosphoglucose isomerase; PGK, phosphoglycerokinase; PGM, phosphoglucomutase; PK, pyruvate kinase; PPDK, pyruvate orthophosphate dikinase; PPT, phosphoenolpyruvate/phosphate antiport; PRK, phosphoribulokinase; Pyr, pyruvate; RCA, RubisCO activase; RPE, ribulose-phosphate 3-epimerase; RPI, ribose-5-phosphate isomerase; Ru1,5P, ribulose-1,5-bisphosphate; RubisCO, ribulose bisphosphate carboxylase; SUS, sucrose synthase; TA, transaldolase; TK, transketolase; TPI, triose phosphate isomerase; TPT, triosephosphate/phosphate antiport; UGP, UDP-glucose pyrophosphorylase

The generation of plastid pyruvate for FA synthesis from sucrose primarily involved sucrose degradation in cytosol, glycolysis and pentose phosphate pathway (PPP) occurring in both cytosol and plastid (Fig. 3a).

It was found that there were only 1.4-fold higher transcripts for sucrose degradation pathway in yellow nutsedge compared to purple nutsedge (Fig. 3b), which was catalyzed either by sucrose synthase (SUS) into uridine diphosphate glucose (UDP-Glu) and fructose (Fru), or by extracellular cell wall invertase (CWINV) or intracellular neutral invertase (CINV) into glucose (Glu) and Fru (Fig. 3a). *SUS* genes in both nutsedge tubers were highly expressed at levels of over 2800 FPKM/protein, which were at least 15-fold higher than C*INV or CWINV* (Fig. 3a, Additional file 1: Table S2), implying that SUS might play an important role as the preferred enzyme in initial sucrose metabolism in nutsedge tubers. This result is consistent with the observation that SUS activities in plant seeds or potato tuber were significantly higher than INV activities [13–16].

Similar patterns of gene expression involved in glycolysis were also present in the two tuber tissues (Fig. 3b). Transcripts for nearly all of glycolytic enzymes in cytosolic or plastidial compartments were at slightly higher or similar levels in yellow nutsedge compared with purple nutsedge (Fig. 3a). Two exceptions were genes encoding cytosolic isoforms of ATP-dependent phosphofructokinase (PFK) and fructose-bisphosphate aldolase (FBA), and plastid fructose kinase (FK), which were over 2.5-fold higher in yellow nutsedge than in purple nutsedge.

Comparing the transcripts for both plastid and cytosol glycolysis revealed some conserved features between the two tuber tissues. The transcript levels were much higher for almost all genes involved in cytosolic glycolysis than their counterparts for plastidial glycolysis (Fig. 3c), except for genes encoding for phosphoglucomutase (PGM) and glucose-6-phosphate isomerase (GPI), where transcripts were distributed in somewhat balanced manner between the two glycolytic pathways. For a complete glycolytic pathway in the two tubers, the glycolytic genes involved in the last seven steps of the glycolytic pathway (from fructose-bisphosphate aldolase (FBA) to pyruvate kinase (PK)) were overall more abundantly expressed in relative to those in early steps. Notably among these genes, those encoding for cytoplasmic glyceraldehyde-3-phosphate dehydrogenase (GAPDH), FBA and enolase (ENO) were significantly highly expressed (> 1800 FPKM). As a result, these data suggested that the cytosolic glycolysis metabolic pathway, particularly the latter steps, was highly active and might produce more carbon precursors toward FA synthesis in tubers. The gene expression patterns of these two species resembled those observed in heterotrophic non-green oil seeds such as castor, safflower and sunflower [17], but differ from those in photoheterotrophic green seeds such as Arabidopsis, rapeseed and soybean [18] and oil-rich mesocarps of oil palm as well avocado that displayed more balanced distribution of transcripts between cytosol and plastid glycolysis [19–21].

Pentose phosphate pathway, a glycolysis bypass process, has been shown to provide carbon sources for pyruvate generation, which also occurred in both cytosol and plastid [22]. However, the overall transcripts for this pathway in cytosol or plastid was similar between the two species (Fig. 3b).

### Significant difference of transcripts for plastid RubisCO bypass was present between two species

**Fig. 4:**
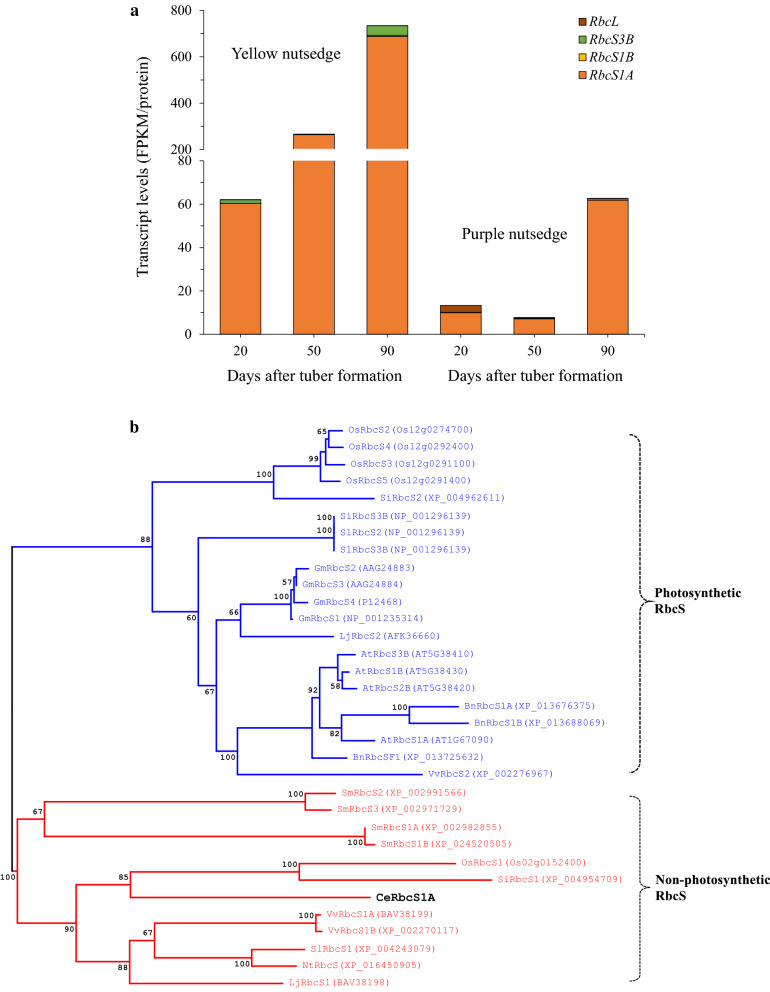

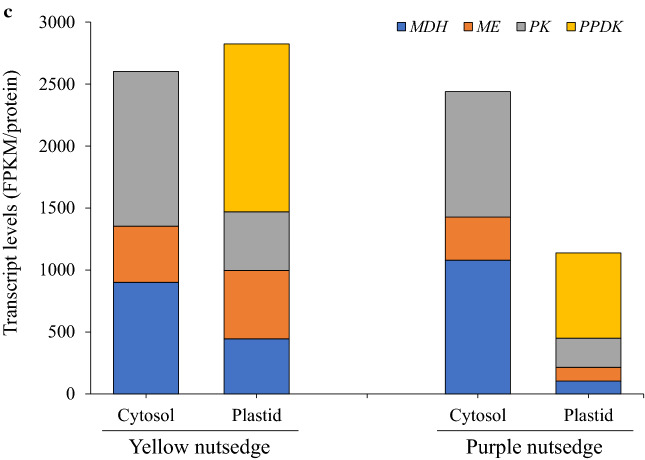
Transcript levels for RubisCO bypass and pyruvate generation. **a** Temporal changes in transcript levels for RubisCO small and large subunits. **b** Phylogenetic tree of tuber RbcS with other plant RbcS isoforms. Phylogenetic analyses were conducted in software DNAMAN v8.0. Trees were constructed using Maximum Likelihood method based on the Jones-Taylor-Thornton (JTT) matrix model. Bootstrap value percentages representing probability of 1000 replicates are indicated at the nodes. The GenBank accession number of each RbcS isoform is shown in bracket. Plants: At, *Arabidopsis thaliana*; Bn, *Brassica napus*; Ce, *Cyperus esculentus*; Gm, *Glycine max*; Lj, *Lotus japonicus*; Nt, *Nicotiana tabacum*; Os, *Oryza sativa*; Sm, *Selaginella moellendorffii*; Si, *Setaria italica*; Sl, *Solanum lycopersicum*; Vv, *Vitis vinifera*. **c** Transcript levels for malate and pyruvate metabolism enzymes

Glycolysis is also bypassed by the production of 3-phosphoglycerate (3-PGA) catalyzed by ribulose-1,5-bisphosphate carboxylase/oxygenase (RubisCO), a key enzyme for fixing carbon dioxide. This process without full Calvin cycle is called Rubisco bypass or shunt [23]. Intriguingly, the expression of genes encoding for RubisCO orthologs also appeared in two nutsedge tubers. In this study, six unigenes encoding for RubisCO small subunit (RbcS) and one for RubisCO large subunit (RbcL, ATCG00490) were detectable to express during tuber development, though RbcL was merely slightly transcribed (Fig. 4a; Additional file 1: Table S2). The presence of *RubisCO* genes, particularly *RbcS* in nutsedge tubers is surprising, since tubers are non-green and non-photosynthetic underground tissues that require little or no light for their development and growth. It is unclear why these RbcS proteins are maintained in the non-photosynthetic tubers, but one can expect that this type of RbcS may be evolved to accomplish different functions other than Calvin cycle occurring in photosynthetic tissues. Orthologs of tuber RbcS were also found in other non-photosynthetic tissues [24, 25]. This type of RbcS was first identified in secretory cells of glandular trichomes of *Nicotiana tabacum* [26] and, therefore, named as T-type RbcS. So far, the T-type RbcS was found to be almost entirely absent in photosynthetic tissues, but essentially expressed in non-photosynthetic tissues of diverse plant species, such as *Oryza sativa* (leaf sheath, culm, anther, and root central cylinder), *Setaria italica* (seed), *Solanum lycopersicum* (stamen, pistil, and green fruit), *Lotus japonicus* (root, nodule, seed, and various floral organs), *Vitis vinifera* (mature leaf and green berry), and *Selaginella moellendorffii* (rhizome and root) [25]. A phylogenetic analysis (Fig. 4b) based on the deduced amino acid sequences revealed that T-type RbcS homologues underwent an evolutionary separation from the photosynthetic relatives, implying that their function may shift when photosynthesis is less active. It was suggested that this type of RbcS might adapt RubisCO to particular environment, for example high CO_2_ concentration generated by intense metabolic pathways in non-photosynthetic tissues that are less permeable to gas exchange [27].

In this study, the *RbcS* genes were transcribed at low levels in purple nutsedge. In contrast, they were abundantly expressed in yellow nutsedge and displayed up-regulation patterns during tuber development (Fig. 4a), with an average transcript level more than 12 times higher than that of purple nutsedge (Fig. 3a). A recent study also showed that the ortholog of *RbcS* in potato starchy tuber displayed much more abundant transcripts in oil-accumulated transgenic lines expressing Arabidopsis *WRI1* gene than in the control [28]. These results match the analysis of the silique of rapeseed, which revealed that high oil content was closely associated with enhanced *RbcS* expression levels and its activities [29]. Evidence has shown that changes in the *RbcS* transcript abundance were directly correlated with the changes in Rubisco level [30, 31]. High expression of *RbcS* genes possibly reflected the high CO_2_ environment [32] and was probably associated with the ability of capturing CO_2_ resulting from the conversion of malate to pyruvate or pyruvate to acetyl-CoA occurring in the plastid catalyzed by NADP-dependent malic enzyme (ME) or pyruvate dehydrogenase complex (PDHC), respectively [15]. Previous studies demonstrated that RubisCO bypass of the Calvin cycle in green oil-rich seeds recaptured CO_2_ brought about less loss of carbon as CO_2_ and produced more 3-PGA, thus improving carbon conversion efficiency toward for fatty acid synthesis [23, 32–35].

### Transcripts for plastid malate and pyruvate metabolism were strikingly distinct between two species

Pyruvate is the important carbon precursor required for plastid fatty acid synthesis. It can be generated either from phosphoenolpyruvate (PEP) catalyzed by PK or pyruvate phosphate dikinase (PPDK), or through the malate metabolism involved in malate dehydrogenase (MDH) and ME that catalyze the sequential production of malate and pyruvate from oxaloacetate (OAA) (Fig. 3a). In this study, the abundant expression of genes encoding for MDH, ME, and PK occurred in both cytosol and plastid, and the transcript level of MDH plus ME was comparable to that of PK (Fig. 4c), implying that the malate metabolism may be as important as PK in pyruvate generation for fatty acid synthesis in nutsedge tubers. Previous studies indicated that malate was a major substrate for fatty acid synthesis in non-green seeds of safflower, castor bean, and sesame [36–38].

Higher expression of MDH, ME, and PK was noted in the cytosol than in the plastid (Fig. 4c), suggesting that the cytosolic pyruvate generation might be more prominent in two species, and malate and pyruvate produced in the cytosol could be transported to the plastid for subsequent pyruvate metabolism for fatty acid synthesis.

One interesting aspect from this comparison was that transcripts for cytosolic MDH, ME and PK were similar in both nutsedge species (Fig. 4c). By contrast, transcript levels for plastid counterparts, along with PPDK, were over two times higher in yellow nutsedge than in purple nutsedge. This suggested that plastid malate and pyruvate metabolism was more active and might produce more carbon source required for fatty acid synthesis in yellow nutsedge relative to purple nutsedge. Therefore, our results implied that up-regulation of genes involved in plastid malate and pyruvate metabolism might play an important role in providing pyruvate for high oil synthesis.

### Transcripts for fatty acid synthesis enzymes in plastid were more abundant in yellow nutsedge than in purple nutsedge

**Fig. 5:**
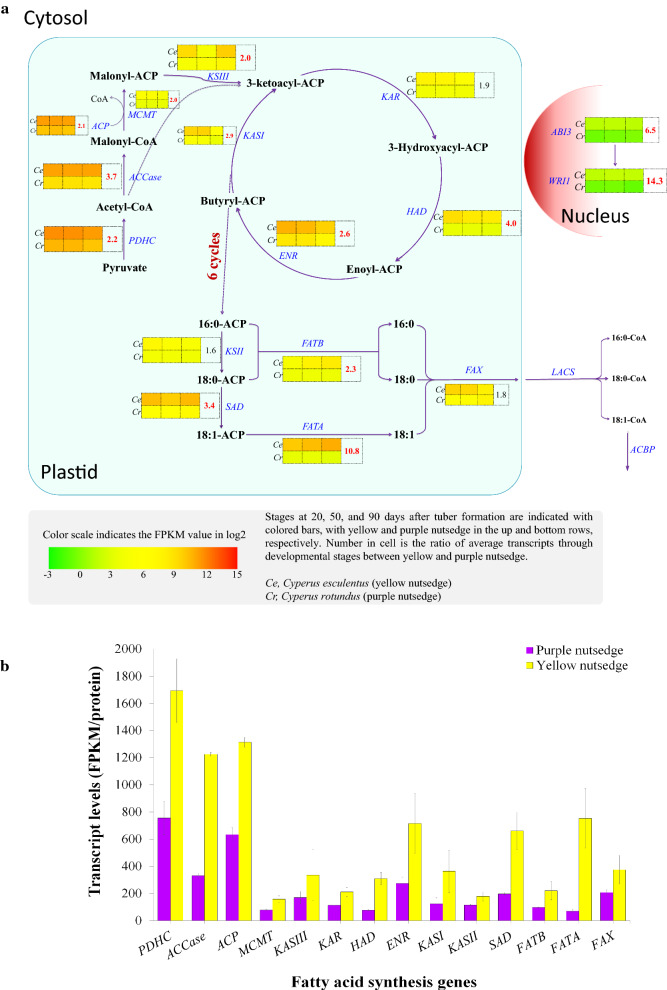
Transcript levels of fatty acid synthesis. **a** Schematic pathway of fatty acid synthesis. Gene names are indicated in blue. Value in table cell indicates the transcript ratio of yellow to purple nutsedge. Ratios more than twofold are showed in red boldface. **b** Average transcript levels for plastid fatty acid synthesis genes. The data are averaged on three tuber developing stages, with error bars indicating standard deviation. ABI3, Abscisic Acid Insensitive 3; ACBP, acyl-CoA-binding protein; ACP, Acyl Carrier Protein; ACCase, acetyl-CoA carboxylase; ENR, Enoyl-ACP Reductase; FAT, Acyl-ACP Thioesterase; FAX, Fatty Acid Export; HAD, Hydroxyacyl-ACP Dehydratase; KAR, Ketoacyl-ACP Reductase; KAS, Ketoacyl-ACP Synthase; LACS, Long-Chain Acyl-CoA Synthetase; MCMT, Malonyl-CoA:ACP Malonyltransferase; PDHC, Pyruvate Dehydrogenase Complex; SAD, Stearoyl-ACP desaturase; WRI1, WRINKLED1

At least fourteen proteins required for de novo fatty acid synthesis from pyruvate in the plastid were all detectable to transcribe in two species (Fig. 5a). Among these proteins, PDHC, acetyl-CoA carboxylase (ACCase), and acyl-carrier protein (ACP) were more abundantly expressed than any other enzymes (Fig. 5b), implicating their important roles in fatty acid synthesis. The overall transcripts for the three proteins accounted for over 50% of the total fatty acid synthesis gene expression at each stage of tuber development. Similar phenomena were also observed in developing oil-rich seeds and mesocarps [17, 19, 21].

Almost all proteins were transcribed at comparatively higher levels in yellow nutsedge, coinciding with high oil accumulation in its tubers. Overall, transcript levels for these plastidial proteins were on average 2.5-fold higher in yellow nutsedge than in purple nutsedge (Fig. 2c). Significant individual differences were represented by ACCase, hydroxyacyl-ACP dehydratase (HAD), stearoyl-ACP desaturases (SAD), and acyl-ACP thioesterase A (FATA), for which their transcripts were more than threefold higher in yellow nutsedge as compared with purple nutsedge (Fig. 5a), suggesting that the metabolic pathways catalyzed by the four enzymes perhaps were much more active in yellow nutsedge.

**Fig. 6:**
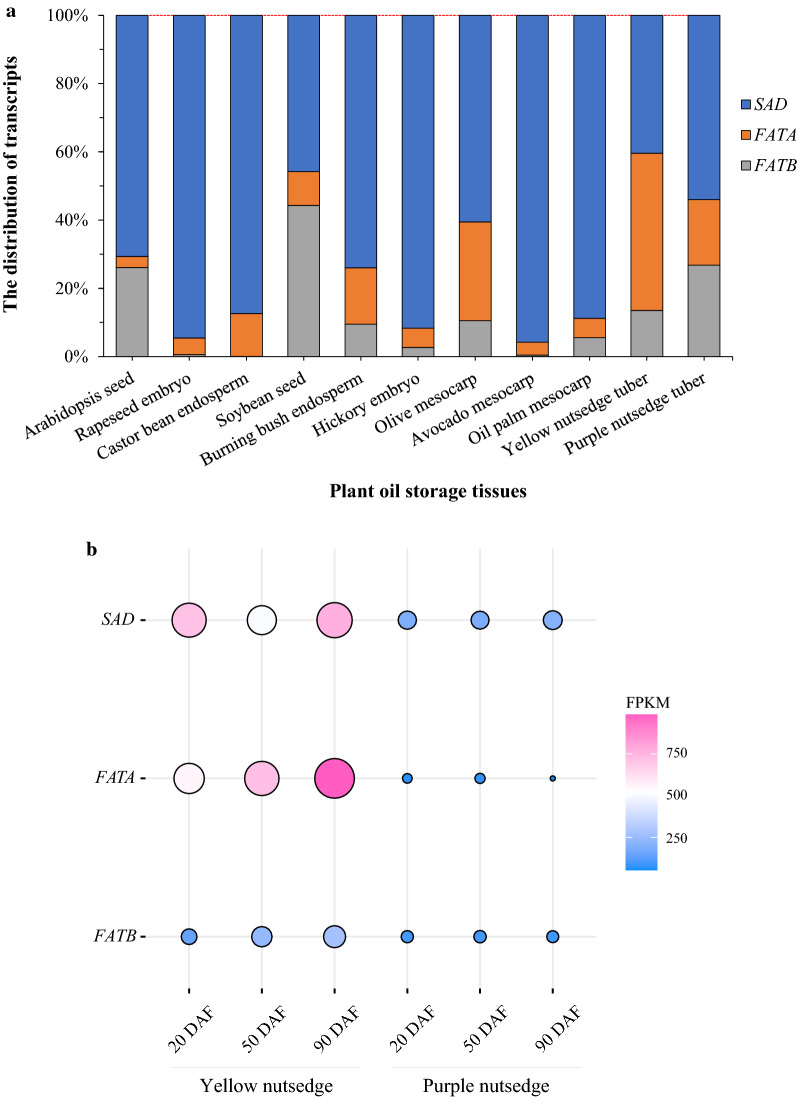
Expression patterns for acyl-ACP desaturase and thioesterase. **a** Relative distribution of transcript levels for SAD, FATA and FATB in plant oil-rich tissues. The data are averaged on all the developing stages of sink tissues. Transcriptome data of various plant species are based on: (1) seeds of Arabidopsis [17], rapeseed [17], castor bean [17], soybean [42], burning bush [17], and hickory [39]; (2) fruits of oil palm [19], olive [41] and avocado [21]; (3) tubers of two nutsedges (this study). **b** Temporal changes in transcript levels (FPKM) for SAD, FATA, and FATB

SAD and FATA are two important enzymes controlling levels of unsaturated fatty acids. Typically, in oil seeds and fruits that rich in unsaturated fatty acids, transcript levels of SAD along with FATA were higher than that of FATB [17, 19, 21] (Fig. 6a). In yellow nutsedge, SAD or FATA were much abundantly transcribed in developing tubers as compared to purple nutsedge (Fig. 6b). In particular, *FATA* genes were up-regulated during tuber development and expressed at more than tenfold higher levels in yellow nutsedge than in purple nutsedge at tuber maturation, correlating with their fatty acid profiles of oil (Fig. 1c). It is noteworthy that *FATA* expression was more than threefold higher relative to FATB in yellow nutsedge, whereas it was lower or comparable to that of *FATB* in purple nutsedge. Intriguingly, high ratio of FATA to FATB transcript was also observed in other plant oil storage tissues rich in oleic acid or its derivatives, such as oil seeds of rapeseed and castor bean [17] as well as hickory [39], and oil fruits of avocado [21] and olive [40, 41]. By contrast, the transcript ratio of FATA/FATB is only 0.1, 0.2, and 1.0, respectively, for seeds of Arabidopsis [17] and soybean [42], and mesocarp of oil palm [19] that contain low levels of oleic acid. Altogether, the expression patterns of *SAD* and *FATA* genes in two tubers might reflect the fatty acid composition, and the ratio of *FATA* to *FATB* expression was likely correlated with the content of C18:1.

### Transcripts for most TAG synthesis genes in endoplasmic reticulum were similar or less in yellow nutsedge than in purple nutsedge

**Fig. 7:**
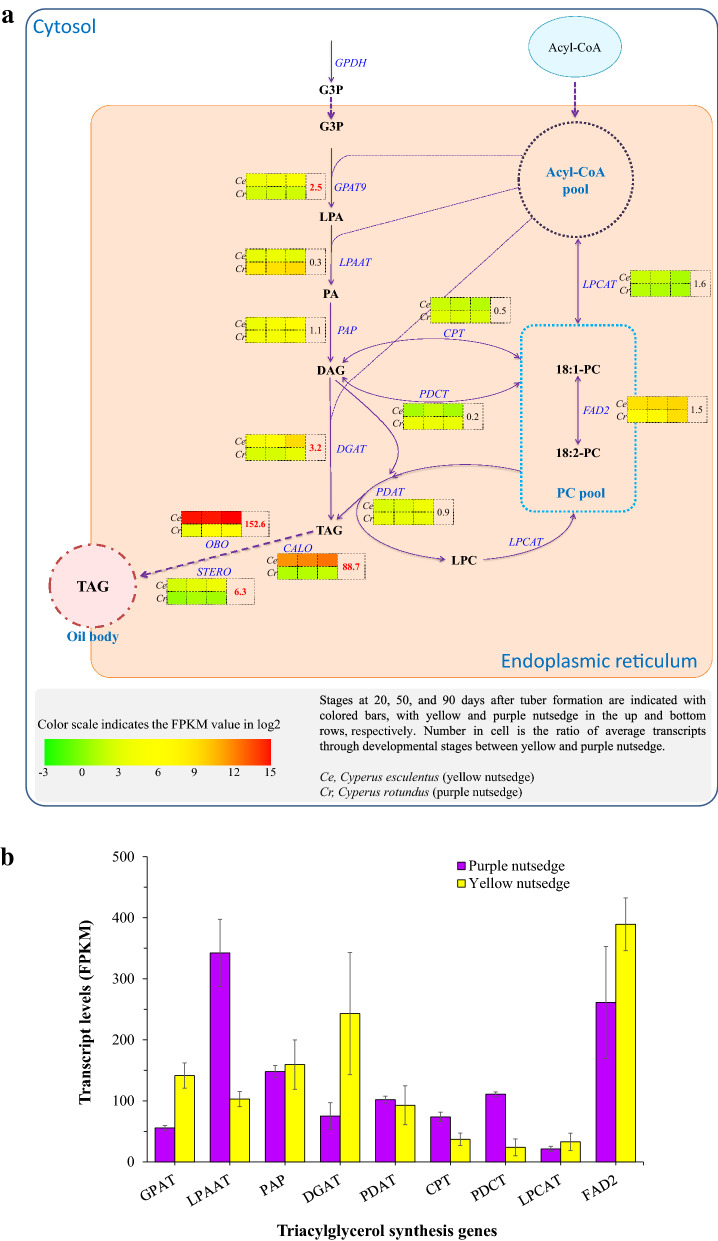

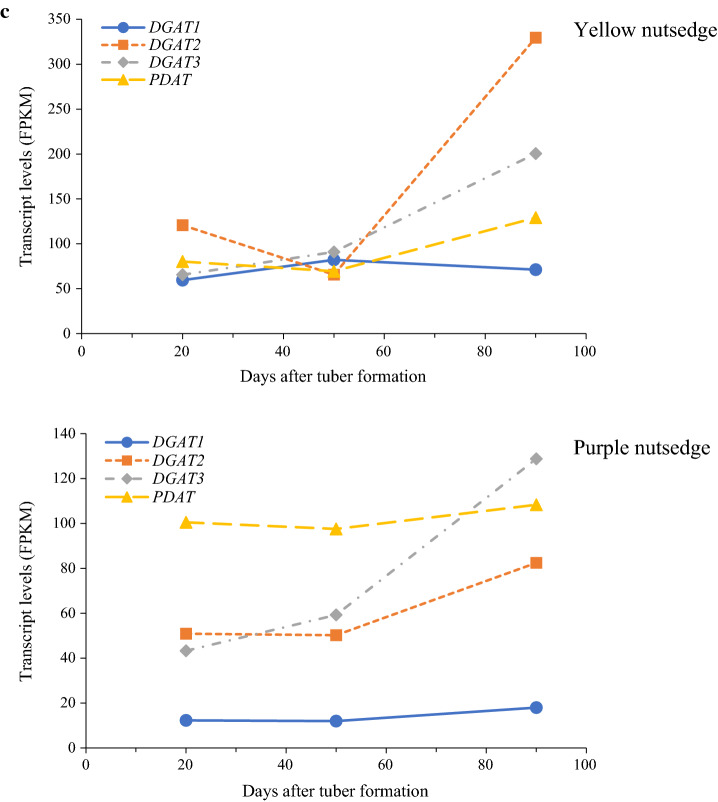
Expression patterns for genes related to triacylglycerol synthesis. **a** Schematic of triacylglycerol (TAG) synthesis pathways. Gene names are indicated in blue color. **b** Average transcript levels for TAG synthesis genes in two nutsedges. The data are averaged on all three developing stages of tubers. The transcript values for subunits of a protein or for multiple isoforms were summed. **c** Temporal transcripts for DGAT and PDAT enzymes during tuber development. CPT, Diacylglycerol cholinephosphotransferase; DAG, Diacylglyceol; DGAT, Acyl-CoA:Diacylglycerol Acyltransferase; FAD2, Oleate desaturase, Fatty acid desaturase 2; G3P, Glycerol-3-Phosphate; GPAT, Glycerol-3-Phosphate Acyltransferase; GPDH, NAD-dependent Glycerol-3-Phosphate Dehydrogenase; LPA, lysophosphatidic Acid; LPAAT, 1-acylglycerol-3-phosphate acyltransferase; LPC, lysophosphatidylcholine; LPCAT, 1-acylglycerol-3-phosphocholine Acyltransferase; PA, Phosphatidic Acid; PAP, Phosphatidic Acid Phosphohydrolase; PC, Phosphatidylcholine; PDAT, Phospholipid:Diacylglycerol Acyltransferase; PDCT, Phosphatidylcholine:diacylglycerol cholinephosphotransferase;

In contrast to transcript patterns for plastidial fatty acid genes, most TAG synthesis genes in endoplasmic reticulum were expressed at similar or lower levels in yellow nutsedge as compared to purple nutsedge (Fig. 7a). For example, phosphatidic acid phosphohydrolase (PAP), phospholipid:diacylglycerol acyltransferase (PDAT), lysophosphatidylcholine acyltransferase (LPCAT) and Δ12-oleate desaturase (FAD2), an enzyme responsible for synthesis of C18:2 fatty acid, displayed similar expression patterns between yellow and purple nutsedge, while lysophosphatidyl acyltransferase (LPAAT) and two enzymes that catalyze fatty acyl exchange between phosphocholine (PC) and diacylglycerol (DAG) through "acyl exchange/editing" processes [43], cytidine-5-diphosphocholine:diacylglycerol cholinephosphotransferase (CPT) and phosphatidylcholine:diacylglycerol cholinephosphotransferase (PDCT), had two- to fivefold lower transcripts in yellow nutsedge than in purple nutsedge. It was demonstrated that acyl editing under the reversible action of PDCT and/or CPT was the major mechanism directing flux of PC-derived polyunsaturated fatty acids such as C18:2 and C18:3 into TAG synthesis [43, 44]. Mutation of *PDCT* gene was shown to bring about a significant decrease in contents of C18:2 and C18:3 in seed oil, with a concomitant increase in C18:1 content [43]. Heterologous expression of flax *PDCT* in Arabidopsis was indicated to increase the levels of C18:2 and C18:3 of seed TAG, while at the same time decrease the proportion of C18:1 [45]. Intriguingly, coinciding with the down-regulation expression of *PDCT* and *CPT* genes in yellow nutsedge, the contents of C18:2 and C18:3 of tuber oil were lower as compared to purple nutsedge (Fig. 1c).

Two exceptions were noted for glycerol-3-phosphate acyltransferase (GPAT9) and diacylglycerol acyltransferase (DGAT) that possessed 2.5- and 3.4-fold higher transcripts in yellow nutsedge than in purple nutsedge, respectively (Fig. 7b). DGAT is the key enzyme that catalyzes the final step of acyl-CoA dependent TAG synthesis [46]. In the two species, transcripts were much higher for DGAT2 than for DGAT1, suggesting a more prominent role of DGAT2 than DGAT1, and DGAT2 may be a key mediator in tuber oil production. Our recent study of DGAT1 and DGAT2 functional analysis has provided an evidence to support this hypothesis [6].

It was noted that PDAT, an enzyme responsible for the transfer of fatty acyl moiety from PC to DAG destined to TAG synthesis, and an ortholog of *Arabidopsis thaliana* DGAT3 (AT1G48300), the soluble cytosolic enzyme that might catalyze TAG synthesis using cytosolic acyl-CoA pool [47], were also detectable to have more abundant transcripts than DGAT1 during tuber maturation (Fig. 7c), but displayed similar expression patterns between the two species. Furthermore, transcripts for DGAT2, DGAT3 and PDAT enzymes in yellow nutsedge were all up-regulated during tuber maturation, coinciding with tuber oil accumulation. Taken together, our data implicated the important roles of DGAT2, DGAT3, and PDAT rather than DGAT1 played in transcriptional regulation of TAG synthesis in the nutsedge tubers.

### Great transcriptional divergence of TAG storage genes between two species

**Fig. 8:**
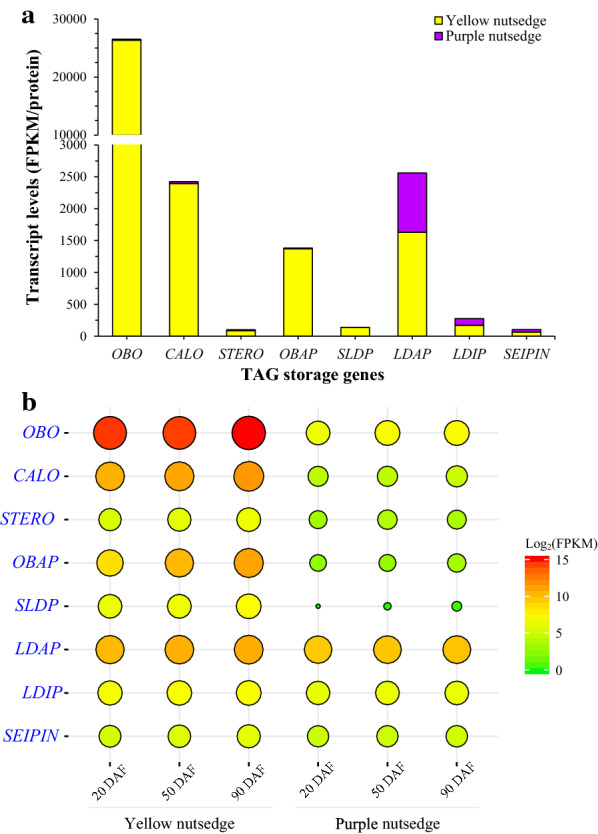
Transcript patterns for triacylglycerol storage proteins. **a** Average transcript levels for diverse TAG storage genes. The data are averaged on three tuber developing stages and the transcript values for subunits of a protein or for multiple isoforms were summed. **b** Balloon plot showing temporal changes in transcript levels (as log_2_(FPKM)) for TAG storage genes. CALO, Caleosin; LDAP, Lipid droplet associated protein; LDIP, LDAP-interacting protein; OBAP, Oil body-associated protein; OBO, Oleosin; SEIPIN, adipose-regulatory protein.; SLDP, Seed lipid droplet protein; STERO, Steroleosin. DAF, days after tuber formation

Similar to oil seeds of plants [17], oil-tuber of yellow nutsedge was represented by the abundant expression of a large number of genes encoding for seed-like oil body proteins such as oleosin (OBO), caleosin (CALO), steroleosin (STERO), oil body associated protein (OBAP), and seed lipid droplet protein (SLDP), with OBO transcript being the most abundant (Fig. 8a, b; Additional file 1: Table S3). In addition, their expression levels increased constantly during tuber maturation, consistent with tuber oil accumulation. However, large difference was noted for these proteins between two species, for which their transcripts were over 6- to 160-fold higher in yellow nutsedge. Similar contrast was also detectable across all the developmental stages (Fig. 8b). Therefore, these results might imply the importance of these oil body proteins particularly oleosin in stabilizing TAG and producing high oil content in yellow nutsedge. Previous studies have shown that oleosin was accumulated in a coincident manner with TAG accumulation and the abundant expression of oleosin was associated with relatively high oil content in seeds [48–53]. For example, overexpression of *OBO* genes in transgenic plant seeds increased oil content by up to 46% as compared to the non-transgenic controls [54, 55].

The high transcripts of oil body proteins in tubers of yellow nutsedge were in sharp contrast to the case in non-seed oily mesocarp tissues of olive, oil palm and avocado, where these structural proteins were poorly transcribed and considered less important to TAG storage or assembly in oil mesocarp tissues [19, 21, 56, 57]. Indeed, other lipid droplet protein such as lipid droplet-associated protein (LDAP) were found to display plentiful transcripts in these oil mesocarps [19, 21].

Intriguingly, a number of genes encoding for LDAP (At3g05500), LDAP-interacting protein (LDIP, At5g16550) and lipodystrophy protein (SEIPIN1, AT5G16460; SEIPIN2, AT1G29760) were also detectable to moderately express in developing tubers of two species (Fig. 8a, b; Additional file 1: Table S3). The transcript levels for LDAP or LDIP were even higher than that of STERO. In addition, the temporal transcript patterns of the three proteins were similar to those of seed-like oil body proteins and in accordance with oil accumulation during tuber development. However, transcripts for the three proteins showed no substantial differences between yellow and purple nutsedge.

It is noteworthy that among these proteins, OBO was most highly expressed in yellow nutsedge, followed by CALO and LDAP in this descending order, similar to the case in oil seeds, where OBO proteins are most abundant on oil body [51]. In purple nutsedge, however, LDAP exhibited highest transcripts, which were five- to 60-fold more abundant than those of other oil body proteins.

Taken together, our results described above indicated that expression patterns of oil body proteins involved in TAG storage were positively associated with oil accumulation, but transcriptional control of these proteins was significantly different between the two species.

### WRI1 and ABI3 showed much higher transcripts in yellow nutsedge than in purple nutsedge

**Fig. 9:**
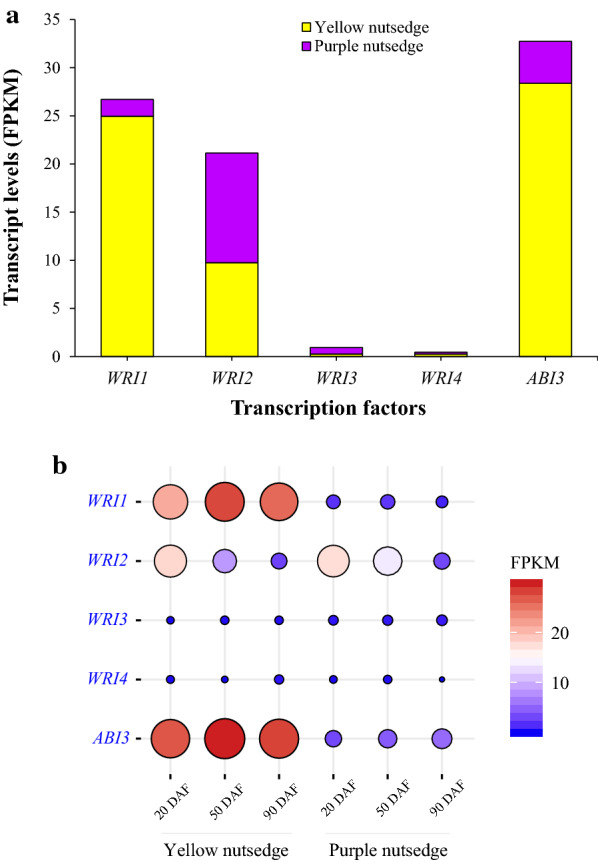
Transcript patterns of WRI1-like and ABI3 transcription factors. **a** Average transcript levels for WRI1-like proteins and ABI3 in nutsedge tubers. The data are averaged on three tuber developing stages and the transcript values for subunits of a protein or for multiple isoforms were summed. **b** Temporal changes in transcript levels (FPKM) for five transcription factors

WRINKLED1 (WRI1) is an important transcriptional regulator in controlling oil accumulation in aboveground oil-rich seeds and fruits of plants, and has been well functionally characterized particularly in the model plant *Arabidopsis thaliana* [58, 59]. In the two nutsedge species, *WRI1* and its multiple orthologs, *WRI2* (AT2G41710), *WRI3* (AT1G16060), and *WRI4* (AT1G79700), belonging to the APETALA2-ethylene responsive element binding protein (AP2/EREBP) family, were all detectable to express in tubers, but they displayed low transcript levels (< 25 FPKM) (Fig. 9a). Particularly, *WRI3* and *WRI4* were only barely expressed, suggesting that they are unlikely to participate in the control of oil production in tubers. This may support the fact that in Arabidopsis, only *WRI1* can activate fatty acid synthesis in oil seeds for oil production, and *WRI3* and *WRI4* are required for cutin synthesis in floral and stem tissues [60]. Although *WRI2* transcript levels were relatively higher compared to *WRI3* and *WRI4*, they were comparable between yellow and purple nutsedge. It has been shown that *WRI2* was unlikely to be associated with fatty acid synthesis [60].

A significant difference between two species was noted for *WRI1* ortholog, which showed on average 14.3-fold higher transcripts in yellow nutsedge over purple nutsedge (Fig. 9a; Additional file 1: Table S3). A similar great contrast was also observed between oil palm and date palm, in which oil palm mesocarp showed 57-fold higher expression of *WRI1* orthologue [19]. In yellow nutsedge, the expression level of *WRI1* was increased with tuber development (Fig. 9b), which was in accordance with oil accumulation pattern in tubers. Furthermore, the temporal expression pattern of *WRI1* matched that of its potential targets such as *PDH-β* (AT2G34590), *BCCP1* (AT5G16390), *FAB2* (AT2G43710), *FATA* (AT3G25110) and *FATB* (AT1G08510).

Unlike oil seed tissues, the maturation master regulators that directly or indirectly control the expression of *WRI1*, such as LEAFY COTYLEDON (LEC1 (AT1G21970) and LEC2 (AT1G28300) and FUSCA3 (FUS3, AT3G26790) [22], were not detectable in two nutsedge tubers, as in oil mesocarps of oil palm and avocado [19, 21], supporting the previous report that FUS3-type proteins are present only in seed tissues, while LEC2-like proteins only appear in dicot plants [61]. Lack of these seed-like regulators suggests that the regulation of WRI1-related transcriptional network in non-seed tissues is different from that of oil seed tissues.

It is noteworthy that in yellow nutsedge tuber, an ortholog of *ABSCISIC ACID INSENSITIVE* 3 (*ABI3*, AT3G24650), the member of B3 domain superfamily, was expressed at comparable levels to *WRI1*, and both of them showed similar temporal expression patterns (Fig. 9a, b). Similar to *WRI1*, *ABI3* ortholog was significantly up-regulated in yellow nutsedge as compared to purple nutsedge. Genes that share similar expression patterns are likely to interact with each other and have regulatory relationships of functionally importance [62]. In this respective, however, it is unclear whether ABI3 is the upstream regulator of WRI1 and controls *WRI1* expression in the nutsedge tubers as in plant oilseeds. Recent study indicated that ABI3 played an important role in regulating plant oil accumulation, which might be independent from WRI1 and LEC2 regulation networks occurred in oil-rich seed tissues [63].

**Fig. 10:**
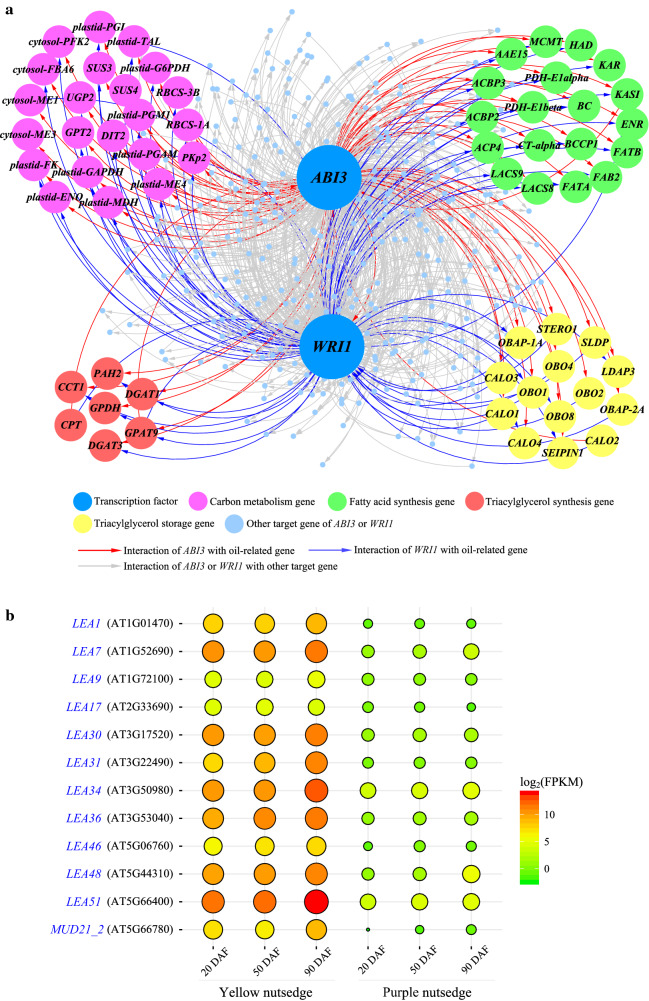
Co-expression network for genes interacting with *ABI3* and *WRI1*. **a** Gene co-expression network of *ABI3* and *WRI1* with selected putative target genes. Genes are represented as nodes and interacting connections are represented as edges that model significant correlation. **b** Balloon plot showing temporal changes in transcript levels (as log_2_(FPKM)) for genes encoding for late embryogenesis abundant (LEA) proteins. DAF, days after tuber formation

To determine the relevant relations between *WRI1* and *ABI3* in regulating oil accumulation in oil tuber, a gene co-expression network was constructed and analyzed using the method of weighted gene co-expression network analysis (WGCNA) [64, 65]. The produced network indicated that these two transcriptional factors were classified as hub genes within a same cluster or module in the co-expression network (data not shown). An interesting finding from the co-expression analysis is that *ABI3* and *WRI1* are co-expressed in concert with the oil genes involved in carbon metabolism, fatty acid synthesis, TAG synthesis, and TAG storage pathways (Fig. 10a; Additional file 1: Table S4). Our analysis result of *WRI1* in concerted regulation with the genes related to carbon metabolism, fatty acid synthesis, and TAG synthesis in yellow nutsedge tuber is similar to recent reports [66, 67]. These oil-related target genes are co-regulated by both of *ABI3* and *WRI1*, indicating an overlapping set of targets for the two transcription factors. It was also shown that transcriptional regulation of *ABI3* and *WRI1* is closely interconnected and *WRI1* is one of direct targets for *ABI3*, suggesting that ABI3 is the regulator of WRI1 in yellow nutsedge, in contrast to oil-rich mesocarps of oil palm and avocado, where WRI1 is not under the control of ABI3 [19, 21]. In oil palm, WRI1 transcriptional activation can be activated by three ABA-responsive transcription factors, NF-YA3, NF-YC2 and ABI5 [68].

It is noted that a large number of genes encoding for late embryogenesis abundant (LEA) proteins, which were charactered well as under control of ABI3 [69–71], were also identified as potential targets of ABI3 and WRI1, as shown in the co-expression network (Additional file 1: Table S4). Much higher transcripts for these LEA proteins in yellow nutsedge than in purple nutsedge reflect the enhanced desiccation tolerance of yellow nutsedge (Fig. 10b), which allows tubers can be stored for long time [8].

Collectively, these results reinforce the fact that WRI1 and ABI3, as the master regulators of oil accumulation, not only regulate gene expressions involved in fatty acid synthesis, but also control the expression of genes encoding for seed-specific oil-body storage proteins [53, 63, 72, 73]. Therefore, WRI1-related regulation network of oil production in yellow nutsedge is most likely to differ either from oil seeds or oil fruits, possibly tuber-specific.

## Discussion

In stark contrast to its close relatives of the same Cyperaceae family such as purple nutsedge, Australian bush onion (*Cyperus bulbosus*), priprioca (*Cyperus articulatus*), water chestnut (*Eleocharis dulci*s) that contain exclusively starch or sugar as the major storage reserves in tuberous sink tissues, yellow nutsedge accumulates high amounts of both oil and starch as the main storage compounds in the tubers. This fascinating feature spur us to carry on the present investigation with the aim to elucidate the molecular mechanism and the specific regulatory factors for oil synthesis in yellow nutsedge that distinguishes it from the carbohydrate-rich relatives. In this study, we have systematically carried out a comprehensive comparative analysis of the global transcriptional profiling of genes involved in tuber oil production between yellow and purple nutsedge that lack genome information publicly available. Also, we presented for the first time a public whole transcriptomic profile and framework for purple nutsedge.

Our results showed that all the key genes responsible for oil production involved in consecutive pathways of carbon metabolism, FA synthesis, TAG synthesis, and TAG storage are detectable to be expressed and functionally conserved in both yellow and purple nutsedge tubers, indicating that these two nutsedges keep a common set of oil-related genes during their evolution, even though purple nutsedge accumulates low levels of oil in its tuber.

Yellow nutsedge accumulates much more oil in tubers than purple nutsedge, suggesting the existence of a species-specific transcriptional network controlling oil production. Indeed, differences of expression patterns for oil-related genes were remarkable between the two species. For example, transcripts for plastidial RubisCO bypass as well as malate and pyruvate metabolism were much more abundant in yellow nutsedge than in purple nutsedge, suggesting that pyruvate availability in the plastid is an important factor for higher oil accumulation. In addition, yellow nutsedge expressed much higher levels of transcripts for almost all fatty acid synthesis enzymes than purple nutsedge, implying that transcriptional regulation of fatty acid synthesis genes is another important factor related to high oil accumulation. Furthermore, the large difference of transcripts for TAG storage genes between two species reflected that TAG packaging and/or stability play a significant role in producing high levels of oil. Interestingly, the above-mentioned differentially expressed genes were regulated in tight coordination with both hub genes of *ABI3 and WRI1*, implying that ABI3 and WRI1 are potential key factors and master regulators for high oil accumulation in yellow nutsedge.

There is growing evidence to show that RubisCO bypass that is not being used in Calvin cycle in plant plays an important role in fatty acid synthesis [23, 31–34]. Enhanced fatty acid and oil synthesis were believed to tightly linked with the increased CO_2_ assimilation through RubisCO shunt when a high CO_2_ environment occurred [74, 75]. In this study, temporal transcripts for RubisCO bypass, particularly *RbcS1A*, were correlated well with oil accumulation in tubers, and were expressed at higher levels in yellow nutsedge than in purple nutsedge (Fig. 4a). These results might reflect that the RubisCO bypass process in yellow nutsedge tuber was more intense and consequently would result in an increase in CO_2_ assimilation. In this context, a higher CO_2_ concentration was most possibly present in non-photosynthetic tuber of yellow nutsedge as compared to purple nutsedge, which may be due to the more active metabolic pathways of plastidial pyruvate and acetyl-CoA formation catalyzed by ME and PDHC enzymes, respectively. Indeed, more abundant transcripts for ME and PDHC were present in tubers of yellow nutsedge in relative to purple nutsedge (Fig. 4c). However, whether the expressions of *RbcS* genes in nutsedge tubers are related to high CO_2_ concentration and whether they are involved in CO_2_ recapture other than photosynthetic CO_2_ fixation remain to be further studied. Nevertheless, the relatively high expression levels of *RbcS* genes in yellow nutsedge compared to purple nutsedge indicated that *RbcS* genes might play a part role in carbon metabolism for oil production in oil-rich tuber tissue.

Oil body proteins have been shown to maintain oil body integrity and protect TAG from coalescence and degradation [53, 74], emphasizing the importance of TAG packaging/stability in oil bodies, which was thought to be rate limiting and a key determinant for oil accumulation [76, 77]. An increasing body of evidence have indicated that oil accumulation appeared to be tightly associated with the expression levels of TAG storage genes, particularly *OBO* genes. The results in this study clearly demonstrated that a large number of TAG storage genes encoding for oil body proteins were highly expressed in tubers of two nutsedges (Fig. 8a, b). These proteins not only involve non-seed lipid droplet associated proteins such as LDAP, but also seed-specific oil body-related proteins including oleosin, caleosin, and OBAP, suggesting that seed-like oil body proteins were co-opted for vegetative tubers during nutsedge evolution. Notably, it was seed-like TAG storage genes rather than non-seed ones that were significantly differentially expressed between the two species and exhibited much more abundant transcripts in yellow nutsedge, implying a major role of them in protecting TAG stability for determining high tuber oil production. TAG stability is vital to enabling oil accumulation and tend to generate an ongoing demand for de novo fatty acid biosynthesis [74, 75]. In view of this, much higher expression of TAG storage genes in yellow nutsedge may induce a "pull" of more carbon into fatty acids, triggering an upstream upregulation of carbon movement into fatty acid substrates such as pyruvate, which in turn promoted CO_2_ recycling. This possibility may be reflected in more abundant transcripts for plastidial RubisCO bypass, pyruvate generation, and fatty acid biosynthesis occurring in yellow nutsedge. Together, our results implicated transcriptional regulation of TAG storage/protection as a major key factor determining high oil accumulation in oil-rich tubers.

Many studies have previously tried to elucidate the molecular mechanism that governs the difference of oil content between high- and low-oil lines in other plant species such as soybean [78], rapeseed [79], maize [80], sunflower [81] and oil palm [82]. Comparative transcriptomic analyses of oil palm with date palm mesocarps [19], and of wild type with transgenic potato tubers expressing Arabidopsis *AtWRI1* [28] have also been conducted to disclose the controlling mechanisms responsible for relatively high or low oil accumulation in these non-seed storage tissues. A common conclusion reached from these studies was that the regulation of gene expression involved in fatty acid synthesis is an important contributor and required for enhanced oil accumulation in diverse plants and tissues. In contrast, our result highlighted that the main point of controlling oil accumulation in oil tuber may well happen not only at the level of fatty acid synthesis, but also at the level of packaging/stabilization of fatty acid in the form of TAG. In other words, the flux of fatty acids to oil body formation may ultimately affect tuber oil content.

## Conclusions

A transcriptomic comparison made in this study for the first time revealed several obviously differential transcript patterns of oil-related genes that distinguish yellow nutsedge from purple nutsedge. Higher oil accumulation in yellow nutsedge against purple nutsedge was strongly correlated with up-regulation of transcripts coding for specific enzymes of plastid Rubisco bypass as well as malate and pyruvate metabolism, almost all fatty acid synthesis enzymes, oil body/lipid droplet proteins, and ABI3- and WRI1-like transcription factors. The most difference is notable for transcripts involved in oil body-related genes. These distinctively expressed genes reflect the differential carbon flux toward oil production between two species and may be the key drivers for high oil accumulation in yellow nutsedge tubers, which not only increased carbon flux to oil production, but also enhanced packaging/stabilizing of TAG into oil bodies. Together, our study represents an important step toward determining the transcriptional control of key genes responsible for the different oil content between two nutsedges and provides a useful reference to explore underlying mechanism leading to high oil production in other oil yielding plant species. In addition, this study provides a molecular basis for metabolic engineering or genetic breeding in the future to enhance oil content in root or tuber crops, or other non-seed tissues through manipulation of the key genes, particularly those related to TAG storage and the master regulator ABI3 as new targets.

## Methods

### Plant materials and growth conditions

The two plant species used in this study are the cultivated varieties of *Cyperus esculentus* L. and *Cyperus rotundus* L*.*, respectively. Undamaged and healthy tubers harvested last year were chosen as seed materials. Before sowing, seed tubers were kept at 4 °C for 4 days and then wrapped in moist blotting paper for 2 days to promote maximum viability and uniform sprouting. After that, tubers were sown in sterilized soil mixture (commercial nutrient soil: vermiculite, 1:1) at 1.0–2.0 cm depth in 22 × 21 cm (diameter × height) plastic pots. Planting density was one tuber per pot. Pots with seed tubers were placed on an experimental plot without any other plants nearby on the condition of natural light and temperature at a location of 116°13ʹ18ʺ E, 39°59ʹ32ʺ N in Beijing, China. Seed tubers were allowed to sprout in moist soil and new plants were grown until maturity. All plants were fertilized biweekly with liquid nutrient Murashige and Skoog (MS) solution [83] and were watered as necessary to keep the soil appropriate moist throughout plant growth period. When needed, plant leaves were clipped after shoot emergence to maintain a height of no more than 50 cm.

The whole plant growth period experienced from mid-April to mid-September of 2016, with climate feature on the average being: April, 10.7 h night at 10 °C/13.3 h day at 22 °C; May, 9.6 h night at 13 °C/14.4 h day at 26 °C; June, 9 h night at 18 °C/15 h day at 31 °C; July, 9.3 h night at 21 °C /14.7 h day at 32 °C; August, 10.3 h night at 20 °C/13.7 h day at 30 °C; September, 11.5 h night at 14 °C/12.5 h day at 26 °C. Humidity during these months is in the range of 32–67%.

Fresh new tubers at different developmental stages were randomly selected and harvested, and then immediately washed and cleaned free of soil and fibrous scaly appendages, and stored in -80 °C before use in subsequent experiments.

### Quantitative analyses of proximate components in tubers

Before analysis, tubers were crushed in liquid N_2_ in a mortar.

Oil content and the fatty acid composition were determined using the method as described previously [6].

Proteins were extracted from tuber samples by homogenizing in ice cold buffer (0.5 mol/L NaCl, 0.001 mol/L EDTA, 1% (w/v) SDS, and 0.02 mol/L Tris–HCl, pH 7.5) and was centrifuged at 15,000* g* at 4 °C for 30 min. Protein in the supernatant was measured by a BCA Protein Assay (Thermo Fisher (China) Scientific Inc., Ltd).

For determination of starch and sugar, powdered tuber samples were homogenized in 80% (v/v) ethanol for 10 min and then centrifuged at 6000* g* for 10 min. The liquid supernatant was used for sugar analysis, while the remaining sediment for starch analysis. Soluble sugars were quantified following the procedure previously used [84]. Starch was measured via the method of two-wavelength iodine binding colorimetry [85].

All the determinations were performed in at least triplicates.

### RNA isolation

Total RNA was extracted according to a protocol [86] modified from CTAB-based method [87]. Pure high-quality RNA samples dissolved in non-RNase water were stored in liquid N_2_ for further analysis (Additional file 2).

### RNA deep sequencing

Before sequencing, the purity, concentration and integrity of RNA samples were assessed using Nanodrop 2000 spectrophotometer, Qubit 2.0 Fluorometer, and Agilent 2100 Bioanalyzer to ensure samples qualified for RNA sequencing. RNA sequencing was carried out on an Illumina Hiseq4000 sequencing and analysis platform (Biomarker Technologies Corporation, China). High-quality reads filtered from generated raw reads (raw data) were de novo assembled using the Trinity program [88]. Annotation for assembly sequences (> 200 bp) was conducted using a BLAST homology search against the NCBI NR database (ftp://ftp.ncbi.nlm.nih.gov/blast/db/), Swiss-Prot (http://www.ebi.ac.uk/uniprot/), GO (http://www.geneontology.org/), COG (http://www.ncbi.nlm.nih.gov/COG/), KOG (ftp://ftp.ncbi.nih.gov/pub/COG/KOG/), eggnog (http://eggnog.embl.de) and KEGG (http://www.genome.jp/kegg/). Transcript levels of genes were quantified based on the number of fragments per kilobase of exon model per million mapped reads (FPKM) [89].

### Gene co-expression network construction

Co-expression network was constructed based on the transcript data sets using the method of weighted gene co-expression network analysis (WGCNA) [64, 65]. Pearson’s correlation coefficient above a threshold of 0.9 for a pair of gene expression was used to filter the gene pairwise connection. The resulting network was displayed with Cytoscape software platform (http://www.cytoscape.org) [90].

## Supplementary Information

**Additional file 1: Table S1.** Summary of transcriptome data sets, assembly and annotation from tuber samples of yellow and purple nutsedge. **Table S2.** Annotation and expression levels for genes associated with carbohydrate metabolism. **Table S3.** Annotation and expression levels for genes associated with lipid metabolism. **Table S4.** Selected genes in tight transcriptional coordination with *ABI3* and *WRI1*. **Table S5.** Primer pairs of selected genes used for qRT-PCR.

**Additional file 2: Fig. S1.** Relative expression levels of selected genes determined by qRT-PCR.

## Data Availability

RNA-seq raw data are available on the National Center for Biotechnology Information (NCBI) Sequence Read Archive (SRA) BioProject under accession number PRJNA671562.
