# Supplementary material for: High oil accumulation in tuber of yellow nutsedge compared to purple nutsedge is associated with more abundant expression of genes involved in fatty acid synthesis and triacylglycerol storage
Source: Biotechnol Biofuels. 2021 Mar 2;14:54. doi: 10.1186/s13068-021-01909-x (PMC7923336; doi:10.1186/s13068-021-01909-x)
Supplement: Supplementary file 2 — Additional file 2: Fig. S1. Relative expression levels of selected genes determined by qRT-PCR. [file 13068_2021_1909_MOESM2_ESM.pdf]

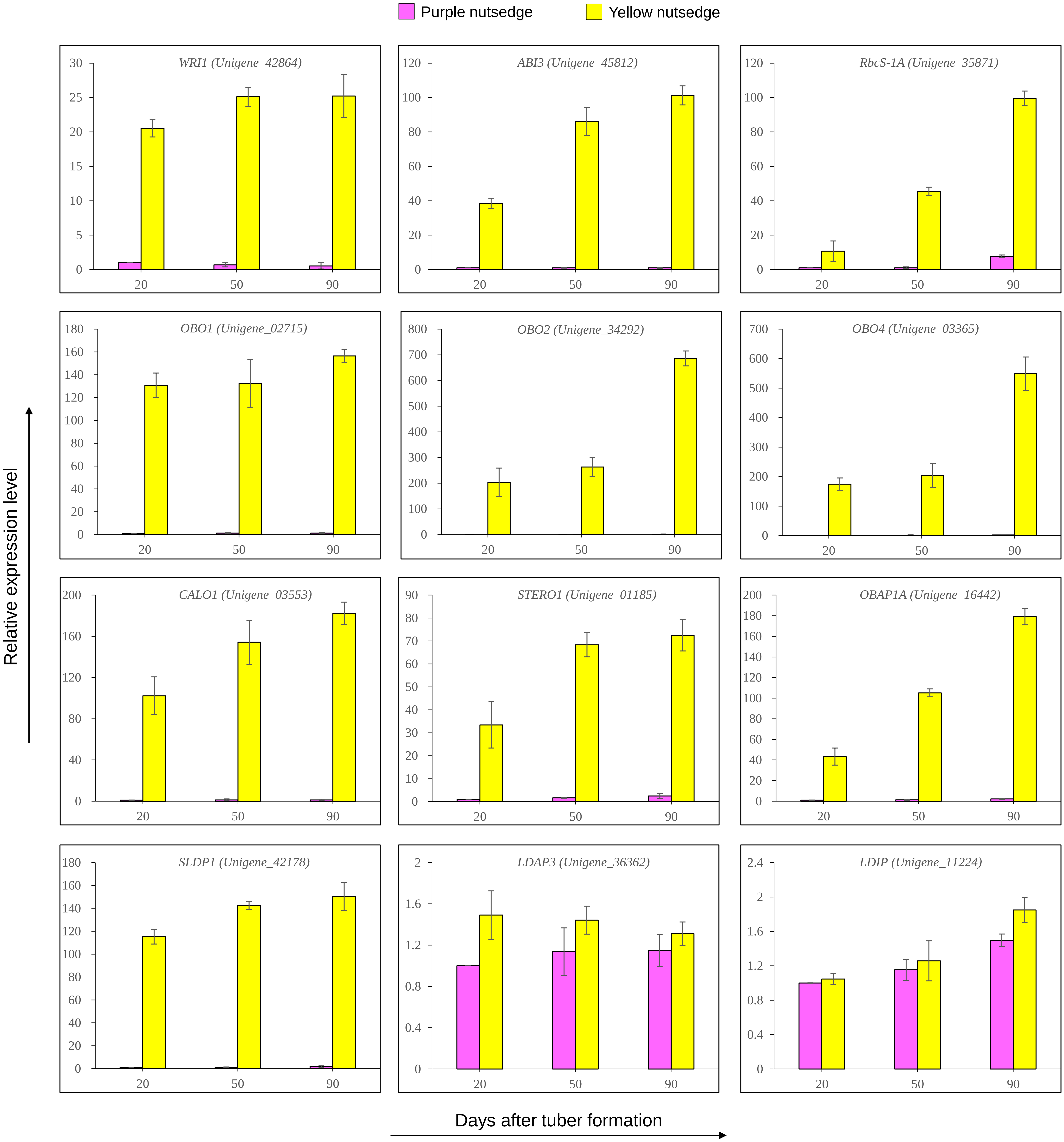

Fig. S1. Relative expression levels of selected genes determined by real time quantitative PCR (qRT-PCR) in at least triplicates following the method as described in [84]. Values represent means±SD.
